# Effect of Enzymatic-based chemomechanical caries removal agent on proliferation and osteogenic differentiation of dental pulp stem cells

**DOI:** 10.1186/s12903-025-07495-w

**Published:** 2025-12-27

**Authors:** Hend A. Gouda, Hamdi H. Hamama, Menatalla M. Elhindawy, Asmaa S. Elmahdy, Youssry M. Elhawary

**Affiliations:** 1 Faculty of Dentistry, Mansoura University, Mansoura, Egypt; 2 Faculty of Dentistry, New-Mansoura University, New-Mansoura, Egypt; 3 Faculty of Oral and Dental Medicine, Alsalam University, Tanta, Egypt; 4 Faculty of Dentistry, Horus University, New-Damietta, Egypt

**Keywords:** Chemomechanical agents, DPSCs, DSPP, Enzymatic-based CMCR, MTT, And wound healing

## Abstract

**Background:**

Chemomechanical caries removal (CMCR) offers a non-invasive alternative to conventional drilling techniques; however, the potential cytotoxic and genotoxic effects of CMCR agents on the dental pulp complex remain underexplored. This study aimed to evaluate the effects of enzymatic-based CMCR on the proliferation and osteogenic differentiation of dental pulp stem cells (DPSCs).

**Methods:**

MTT assay, scratch assay, and gene expression analysis were conducted to assess cell proliferation (viability percentage), migration (wound healing percentage), and osteogenic differentiation (RT-qPCR). The viability of DPSCs after direct exposure to enzymatic-based CMCR (0.5%, 1%, and 2% in DMEM) was measured at 24 and 48 h post-exposure. Indirect exposure was also tested using sound mineralized (0.5 mm and 1 mm) and demineralized (0.5 mm) dentin discs. Scratch assay was performed for control, direct, and indirect groups at 0, 24, and 48 h, followed by fixation and crystal violet staining at 48 h. Dentin sialophosphoprotein (DSPP) gene expression was quantified using RT-qPCR after 48 h.

**Results:**

Two-way ANOVA of proliferation and migration assays revealed that both indirect (0.5 mm demineralized dentin) and direct (0.5% DMEM) exposure groups showed the lowest cytotoxic effect after 24 h (82.75 ± 3.37 and 68.11 ± 4.99, respectively). After 48 h, a recovery trend in DPSC viability was observed, with both groups exhibiting the highest proliferation (93.60 ± 4.67 and 78.25 ± 4.88, respectively). These groups also showed the highest wound closure percentages (78.78 ± 0.89 for direct and 74.19 ± 1.03 for indirect exposure). One-way ANOVA of RT-qPCR results demonstrated significant upregulation of DSPP gene expression in the indirect (0.5 mm demineralized dentin) group (5.35 ± 2.01, *p* < 0.05).

**Conclusion:**

Enzymatic-based CMCR exhibits favorable biocompatibility, particularly when applied directly on DPSCs at low concentrations and when used indirectly through dentin discs. These findings suggest that this enzymatic caries removal approach is potentially safe, effective, and minimally invasive, making it suitable for conservative restorative dentistry.

**Supplementary Information:**

The online version contains supplementary material available at 10.1186/s12903-025-07495-w.

## Introduction

Dental caries has encompassed every part of our globe indicating its significaht spread of this pandemic disease [1].The minimally-invasive removal of dental caries has been extensively applied over the previous 10 years [2]. Once dental caries influences the tooth structure, conservative procedures should be considered to prevent its progress and to minimize the wear of sound tooth structure [3].

Conventionally, caries removal and cavity preparation rely on high-speed rotary instruments and burs, which efficiently remove infected dentin but often result in unnecessary loss of sound tissue, heat generation, and patient discomfort [4]. In recent years, newer minimally invasive approaches have been proposed, including hand and micro-excavators with ultra-fine tips and the use of dental operating microscopes that enhance visualization and precision during caries removal. These techniques promote conservative tissue preservation and improved control over the excavation process. However, they present limitations such as longer operation time, operator dependency, and restricted field of view when using magnification systems. Moreover, chemical caries removal methods for instance, papain-based agents (Papacarie) have been introduced to selectively degrade the infected dentin [5]. This integration reflects the contemporary shift toward minimally invasive, biologically based dental practice [4, 6].

In CMCR technique, the damaged dentin is chemically-softened using chemical agents and then undergoes removal with hand instruments [7, 8]. Importantly, no specific instruments are required for the CMCR, making it an effective, practical, accessible and cheap technique for caries removal. CMCR causes little pain and is often more accepted by patients than other conventional methods [9, 10]. It also reduces the necessity for local anesthesia use and is associated with less accidental exposure of the pulp. So, CMCR has become amore acceptable technique, especially for children, handicapped people and severely anxious patients [4]. Previous studies [11–13] were conducted to evaluate the bonding restorative protocols following caries excavation using CMCR and their outcome recommend utilizing either universal or resin-modified-based adhesives. These adhesive protocols might help in remineralization and strengthening relatively weak caries-affected ‘leathery’ dentin [7].

Enzymatic-based CMCR consists of papain, chloramine, toluidine blue, salts and thickening vehicle, which has bacteriostatic, bactericidal, and anti-inflammatory activities [4, 14]. Papain, derived from the latex of papaya leaves and fruits (Carica apaya), has long been utilized to heal wounds because it has a chemical debridement capacity and stimulates the formation of granulation tissue and promote epithelialization. Chloramine has bactericidal and antiseptic activities, and is able to smooth decayed dentin, easing its removal. The current scientific evidence-based reveled that the shortest cries excavation time is associated with conventional rotary excavation, followed by enzymatic-based CMCR method [5, 7]. Moreover, it has been reported that the longest excavation time is associated with NaOCl-based CMCR technique [5].

Hence, our study investigated the biological effects of enzymatic-based CMCR on DPSCs. The null hypothesis is that CMCR has no side effects on DPSCs’ proliferation and osteogenic differentiation.

## Materials and methods

### Study type and ethical criteria

The ethical committee of Faculty of Dentistry, Mansoura University approved this in vitro study (code no: A02020822). The flowchart of the entire manuscript of this invitro study is delineated in supplementary file 1.

### Sample size justification

Sample size was justified depending on ANOVA test to compare between the groups using G power program (version 3.1.9.4). The total sample size was 45 (each group = 15) which was adequate to determine a large effect size (f) = 0.4876405, (mean values = 16.2, 26.7, and 29.9 for CBG, PG and BG, respectively, and the SD was 12 in each group) [15] with the actual power (1-ß error) was 0.8 (80%) and the significance level (alpha error) was 0.05 (5%) for two-sided hypothesis test.

### Isolation and characterisation of DPSCs

In this study, hDPSCs were isolated from the coronal portion of a healthy permanent third molar extracted for orthodontic reasons from healthy donor aged 18–25 years. A 3% sodium hypochlorite solution was used to disinfect (for 120 s) the extracted tooth. Then, the tooth was rinsed with phosphate buffer saline (PBS) and underwent drying on a sterile cotton gauze. For pulp chamber exposure, a dental diamond fissure bur (Mani, Inc. US) with a high-speed hand piece (NSK, US) was utilized to make a horizontal cut around the cemento-enamel junction. This was done under copious water cooling. After that, the tooth was transferred to transport media composed of basic medium Dulbecco modified essential medium/F12 with L-glutamine (DMEM/F12) (Life science, UK) supplemented with 20% fetal bovine serum (FBS) USA origin (SIGMA, Merck KGaA, Darmstadt, Germany) Cat. No. F2442, penicillin (100 µg/mL) and streptomycin (100 µg/mL) (Biowest, USA). In the biohazard laminar flow hood, all procedures were aseptically performed in 100 mm petri plate (Sterilin).

Sterile endodontic H-file #30 (MANI, Inc, USA) was used for each tooth to remove dental pulp tissue. The latter underwent mincing and was cultured in T-25 flask (Greiner Bio-one, Germany) that contained DMEM/F12 culture media. Incubation was done in an incubator with 5% CO2 and was assessed every day using the inverted microscopy (Olympus Corp, USA) to notice any contamination and cell outgrowth. Cells were harvested when the cultures reached ≈ 80% confluency. Cultures were washed (2 times) with phosphate buffer saline, trypsinized for 2–5 min with 0.05% Trypsin-EDTA (Biowest, US), neutralized by DMEM/F12 containing 10% FBS. In a falcon tube, detached cells were collected, centrifuged at 226 x g for five minutes. Cells were resuspended either in growth medium or were frozen in a medium composed of 90% FBS and 10% DMSO in liquid nitrogen (−196 °C) till being analysed.

#### Characterization of DPSCs

In order to evaluate the surface molecular expression of DPSCs, 5 × 10^5^ third-passage cells were fixed with 4% paraformaldehyde over 15 min. After washing with PBS, cells were labelled with primary antibodies CD105, CD45, CD90 and CD434 for 1 h, then with fluorescein isothiocyanate-conjugated secondary antibodies in darkness for 45 min. Cells that showed CD90 and CD105 positive staining, and that with CD34 and CD45 negative staining were evaluated using FACS caliber flow cytometry (BD Immunocytometry Systems) [16].

### Preparation of dentin discs

At the outpatient clinic, fifteen healthy human permanent premolars with no caries (*N* = 15), in the age group of 18–25 years, were extracted due to orthodontic reasons. Teeth included in the study were mounted in self-curing acrylic resin blocks with their roots inside the blocks and crowns completely outside. The occlusal thirds of crowns were removed. Then, teeth had their crowns sectioned mesiodistally to obtain dentin discs using a precision saw (PICO 155 Precision Saw) with copious water cooling equipped with a 0.5-mm-thick diamond disc, and then the precision saw equipped with a 1-mm- thick diamond disc (Buehler Ltd). Discs were ground using 400- and 600-grit abrasive paper (T469-SF-Norton; Saint-Gobain Abrasivos Ltda., Jundiai, SP, Brazil) to reach a thickness of 0.5 mm and 1 mm. They were evaluated using a digital caliper (Mitutoyo Sul America Ltda. SP, Brazil) to make sure that the desired thickness was reached. The final number of dentin discs were 45 discs (15 discs per group) [17] (Table 1). All discs were cleaned with ultra sonic cleaner using distilled water for 5 min to remove any debris and the smear layer [18, 19].

**Table 1:** Total dentin discs used in each test (Indirect groups)

Tests	Dentin discs	Total
MTT Assay	5 discs (×3 groups)	15
Cell migration assay	5 discs (×3 groups)	15
Gene expression analysis	5 discs (×3 groups)	15

#### Demineralization of dentin discs

For simulation of an artificial caries in dentin, the fifteen dentin discs (thickness = 0.5 mm) were randomly taken out from the phosphate buffer saline and were partially demineralized using an orthophosphoric acid gel 37%, (PH 0.7) for 5 min. Then, discs were rinsed with distilled water until the acid removed [20].

#### Verification of demineralization process by energy-dispersive X-ray spectrometry (EDS)

The demineralization of dentin discs was assessed by chemical analysis produced by Scan Electron Microscopy (SEM) with an EDS (Oxford, 51-ADD0048, Cambridge, UK). The EDS separates the characteristic X-rays from various elements in an energy spectrum, allowing for elementary microanalyses of specimen. Out of these elements, calcium and phosphorus were statistically analyzed. For each disc, we evaluated 4 different points of the dentin surface [21].

#### Sterilization of dentin discs

All dentin discs were immersed in 0.5% chloramine-T for one hour, to ensure that dentin discs were not contaminated by any living microorganisms including bacteria, yeasts, and viruses [22].

### Protocol of application of enzymatic-based CMCR

In this study, a commercially available papain-based CMCR gel (Papacarie Duo™, FGM Dental Products, Joinville, SC, Brazil; Lot No. [L:9703]) was used according to the manufacturer’s protocol. Enzymatic-based CMCR was directly and indirectly applied to DPSCs. In direct method the material was applied with different concentrations. They were obtained by serial dilution in Dulbecco’s Modified Eagle Media (DMEM) without FBS and were vortexed for 5 min to obtain the needed concentrations [23]. In indirect method, the material was applied on different thickness of dentin discs following the protocol of its clinical application without copious irrigation. After 2 min it was wiped using sterile gauze. Then, the dentin discs were cultured separately in 12-well plate with DMEM/F12 without FBS for 24 h. After that, the media of each group was added to DPSCs.

The composition and main functional components of the enzymatic-based CMCR™ material (Papacarie) used in this study are summarized in (Table 2) to clarify its enzymatic and auxiliary constituents.

**Table 2:** The enzymatic-based CMCR™ material (Papacarie) component

Component	Function/Role	Description
Papain	Proteolytic enzyme	A natural enzyme extracted from papaya latex (Carica papaya); selectively digests partially degraded collagen in infected dentin, facilitating its removal without affecting healthy tissue.
Chloramine	Antiseptic agent	Provides bactericidal and bacteriostatic action; disinfects the cavity and helps prevent bacterial growth.
Toluidine blue O	Dye/Antimicrobial indicator	Serves as a coloring and antibacterial agent; helps visualize the treated carious dentin.
Salts (sodium chloride and others)	Osmotic balance	Maintain ionic stability and support the activity of the enzyme and antiseptic components.
Carbopol (polyacrylic acid)	Thickening agent	Provides a gel-like consistency for controlled application and prolonged contact with the carious dentin.
Deionized water	Solvent	Acts as a medium for the dispersion and stability of all components.

### Study design

In the beginning of this invitro study, MTT assay was utilized to assess DPSCs viability, and the study groups were divided as follows:

**Group 0 (Media group) (Negative control group): **DMEM only.

**Group I (DPSCs group) (Positive control group): **DPSCs (10^6^ ) only.

**Group II (Direct groups) (Enzymatic-based CMCR / DPSCs groups).**

**Group II (Direct groups) (Enzymatic-based CMCR/DPSCs groups)**.

- GII. A: The enzymatic-based CMCR concentration at 0.5%.
- GII. B: The enzymatic-based CMCR concentration at 1%.
- GII. C: The enzymatic-based CMCR concentration at 2%.

**Group III (Indirect groups) (Enzymatic-based CMCR/Dentin disc/DPSCs groups)**.

- GIII. A: Demineralized Dentin Discs (DDD) (0.5 mm thick).
- GIII. B: Mineralized Dentin Discs (MDD) (0.5 mm thick).
- G III. C: Mineralized Dentin Discs (MDD) (1 mm thick).

**Fig. 1:**
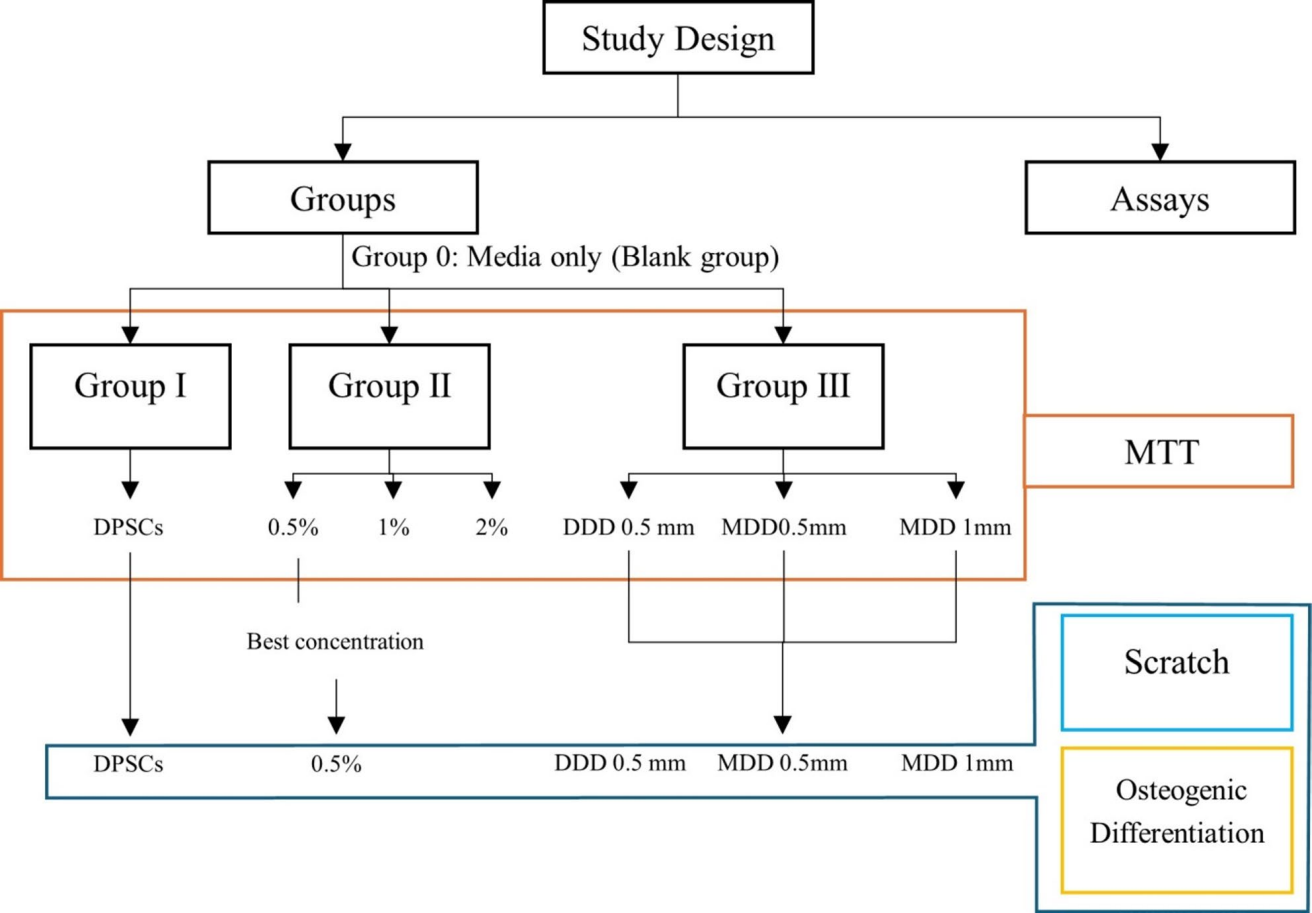
Diagrammatic chart shows study design and study groups for each assay

After MTT assay in the direct groups, the best concentration was selected to continue in scratch and gene expression assays. Figure 1 shows the study design and study groups for each assay.

### Cell viability assessment (MTT Assay)

Cell viability was tested for DPSCs in different groups and was compared with the positive control group (DPSCs). DPSCs were inoculated into each well of 96- microwell plates and underwent 24 h incubation for achieving a “complete cell adherence”. The enzymatic-based CMCR was added according to the protocol of application of each group (GII and GIII) and incubated for 24 h and 48 h. The MTT assay was utilized to measure the number of viable cells. The medium was replaced with MTT (0.2 mg/ml) dissolved in DMEM (Gibco^®^, US) Cat. No 11330057), and incubation was done for additional four hours at 37˚C. DMSO (Sigma-Aldrich Canada Co) Cat. No. D8418 solvent was used to dissolve the formazan product, and the absorbance of lysate, which indicates the activity of mitochondria, was measured at 540 nm by the Multiskan microplate reader (Biochromatic Labsystem, Osaka, Japan) [24].

### Scratch assay

The DPSCs (5 × 10^3^_−_7 × 10^3^) underwent seeding in 6-well plates (Corning, US) in complete media and underwent 48 h- incubation at 37 C. Scratches were performed using a 200 μm pipette tip, and the cell surface was scraped in a straight line guided by the cover. The best concentrations from direct groups (0.5%) and indirect groups were applied for 48 h. Slides were photographed at 0, 24 and 48 h, by Olympus^®^ digital camera installed on Olympus^®^ microscope with 1/2 X photo adaptor, using 40X objective. On Intel^®^ Core I7^®^ based computer, microscopic images underwent analysis by VideoTest Morphology^®^ software (Russia) with a specific built-in routine for area, and % area measurement at 0, 24 h and 48 h. For each well, three predefined regions along the scratch line were analyzed, and the mean area was used for quantification.

The percent wound closure was calculated for each group separately according to the following formula:

$$Percent\;wound\;closure\;\%\;\frac{A0\;-\;At}{A0}\times\;100$$

where A_0_ is the initial wound area at 0 h and A_t_ is the wound area after treatment (24 h and 48 h). After 48 h, the DPSCs of each group was fixed and stained with Crystal violet dye solution (Merck KGaA, Darmstadt, Germany) [25].

### Osteogenic differentiation analysis

**Fig. 2:**
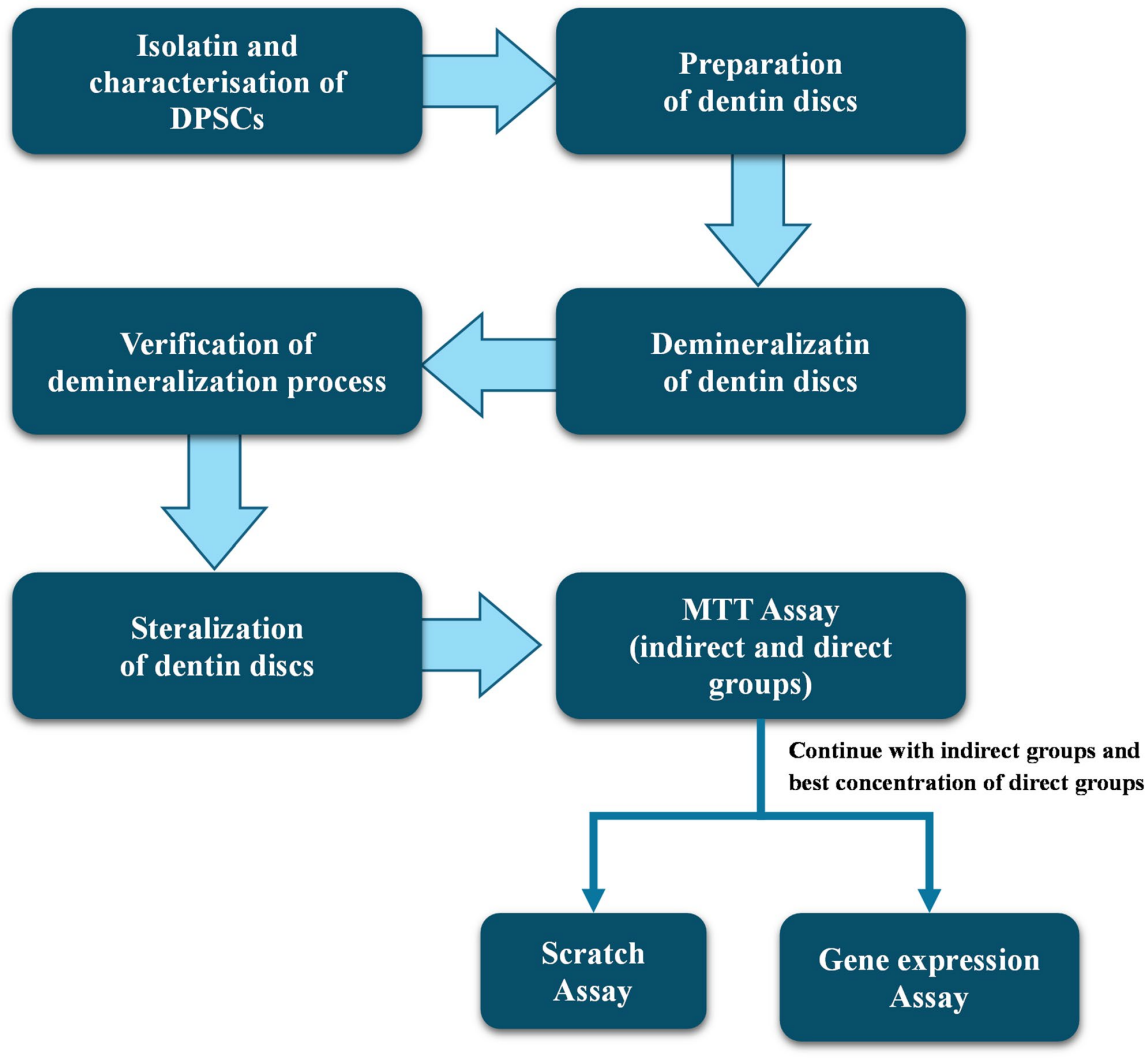
Flow chart shows the methodology

The gene expression for the 0.5% concentrations of enzymatic-based CMCR accompanied with the indirect groups, real-time quantitative PCR (RT qPCR) was performed to determine the control negative (DMFM media) and control positive (osteogenic media) groups. The RNA of DPSCs was extracted with (RNeasy Mini Kit (50) (LOT #: 163046464, QIAGEN GmbH, Hilden, Germany). A High-Capacity cDNA Reverse Transcription Kit (LOT #: 00886149, Thermo Scientific, Baltic, Lithuania) was utilized to convert the extracted RNA (Total RNA) to complementary DNA using a High-Capacity cDNA Reverse Transcription Kit (LOT #: 00886149, Thermo Fisher Scientific, Baltic, Lithuania). Then, all complementary DNA were stored at −20 ͦ C. The qPCR reaction mixture (20 µl), performed three times, contains 2 µl of forward and reverse primers of DSPP gene (Vivantis, Subang Jaya, Malaysia), 2 µl of cDNA template, and 10 µl SYBR green PCR master mix (Thermo Scientific, Lithuania). Amplification was done for 40 cycles. To normalize the cycle threshold (Ct) value for the target gene, the Ct value of control gene (β-actin gene) was utilized [26]. The list of forwarding and reverse primers of selected genes was displayed in (Table 3). Target gene expression was determined by the delta–delta Ct method[27] (Fig. 2).

**Table 3:** The list of forwarding and reverse primers of selected genes

	Gene Symbol	Primer sequence	Type	Chromosome location	Ensembl number
1	**DSPP**	**5-TTAAATGCCAGTGGAACCAT-3**	**Forward**	**4q22.1**	ENSG00000152591
		**5-ATTCCCTTCTCCCTTGTGAC-3**	**Reverse**		
	**β-actin**	**5-CTCTTCCAGCCTTCCTTCCT-3**	**Forward**	**7p22.1**	ENSG00000075624
		**5- AGCACTGTGTTGGCGTACAG-3**	**Reverse**		

### Statistical analysis

All experimental conditions were conducted in triplicate and assessed as 3 independent experiments. Data was represented as means ± SDs. The homogeneity of variance and normal data distribution were tested by Shapiro Wilk test for normality. SPSS software (IBM Corp, US) was utilized for data analysis. Data of proliferation and scratch assays were analysed by Two-way ANOVA while that of osteogenic differentiation were investigated by one-way ANOVA. Each ANOVA test was followed by Tukey’s post hoc multiple comparison test. The significance of a result was set at *p* < 0.05.

## Results

### Isolation and characterization of DPSCs

**Fig. 3:**
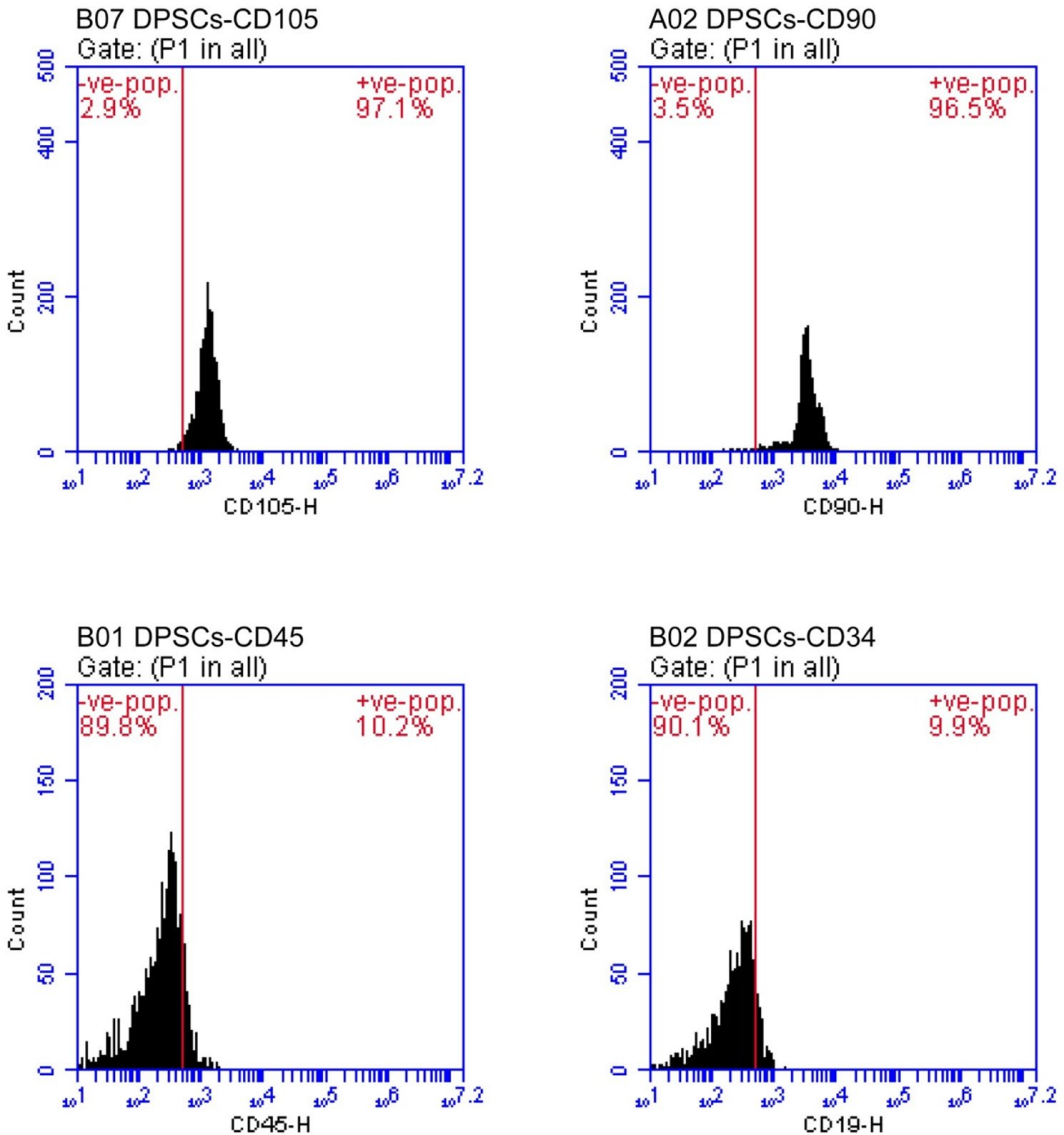
Flow cytometery immunophenotypic characterization of DPSCs histogram shows: positive (+ ve) expression of mesenchymal markers CD105, CD90, and negative (-ve) expression of mesenchymal markers CD45 and CD34

Isolated DPSCs were spindle-shaped. Flow cytometry was utilized for characterization of the phenotype of the third-passage cells **(**Fig. 3**)**. The present data revealed that cells expressed the mesenchymal stem cell markers CD90 (96.5%), and CD105 (97.1%) but did not express hematopoietic markers CD34 (9.9%), and CD45 (10.2%).

### Verification of demineralization process by (EDS)

**Fig. 4:**
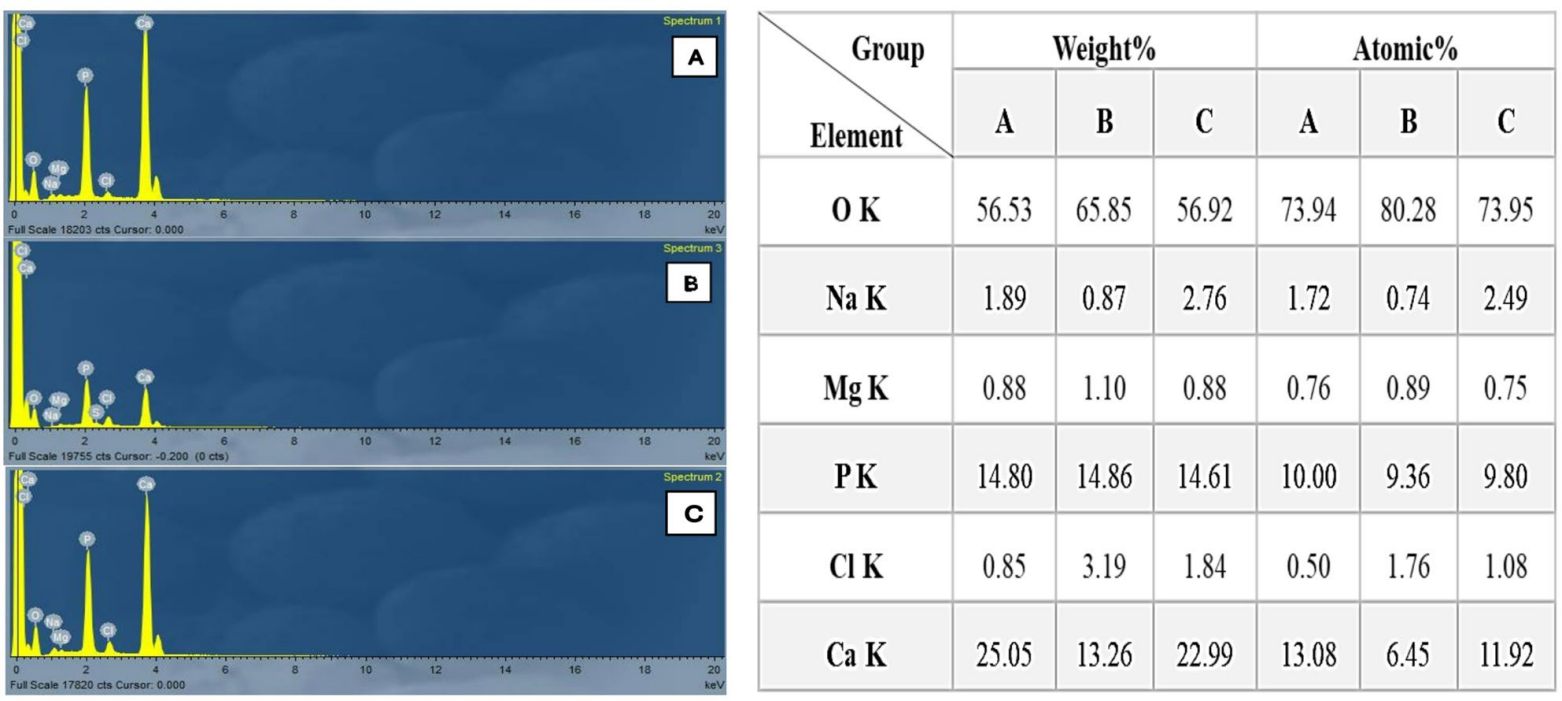
A photograph shows EDS analysis, (**A**): control group (sound discs), (**B**): demineralized group and (**C**): treatment group with enzymatic-based CMCR

EDS analysis demonstrated changes in calcium content after demineralization in comparison to their control and treated counterparts control and treated group with enzymatic-based CMCR groups showed high levels of calcium content. However, the demineralized group showed nearly a 50% lower level of mineral components (Fig. 4).

### Cell viability assay (MTT Assay)

The effects of varying concentrations of enzymatic-based CMCR on DPSCs viability were assessed after 24- and 48-h post-exposure. The cell viability and proliferation between groups regarding exposure (direct and indirect), time (24 and 48 h) and concentration was evaluated by the two-way ANOVA test. Significant differences existed between the study groups (*p* < 0.05). At 24 h, a significant decrease of DPSCs viability (*p* < 0.05) was observed in all experimental groups except indirect 0.5 mm thick DDD. At 48 h, all groups exhibited a trend toward recovery in cell proliferation. Enzymatic-based CMCR at 0.5% direct exposure group exhibited the most significant increase in cell viability and proliferation while higher concentrations (1% and 2%) resulted in pronounced cytotoxicity. Thus, 0.5% concentration was the optimum direct exposure concentration in our study. (Table 4) (Supplementary file 3).

**Table 4:** MTT assay results for hDPSCs cells exposed to different concentration of enzymatic-based CMCR

MTT Assay						
Groups Time	**Direct Exposure (Concentration)**			**Indirect Exposure (Thickness of dentin discs)**		
	**0.5%**	**1%**	**2%**	**Indirect 0.5 mm DDD**	**Indirect 0.5 mm MDD**	**Indirect 1 mm MDD**
After 24 h	68.11 ± 4.99 ^CDEF^	61.87 ± 2.71 ^EF^	53.52 ± 1.95 ^F^	82.75 ± 3.37^ABC^	74.26 ± 4.21^BCDE^	72.13 ± 10.35^BCDE^
After 48 h	78.25 ± 4.88 ^BCD^	76.04 ± 2.97 ^BCDE^	66.68 ± 1.42 ^DEF^	93.60 ± 4.67^A^	85.60 ± 6.86^AB^	80.62 ± 5.50^ABCD^

Values are represented in means ± SDs. Different letters indicate significant differences between groups (*p* < 0.05). Test used: Two-way ANOVA test comparing between the studied groups in different exposure method, time periods and concentration. Values are represented in means ± SDs. Different letters indicate significant differences between groups (*p* < 0.05). Assay is the experimental unit of our study, number = 3.

### Scratch assay

**Fig. 5:**
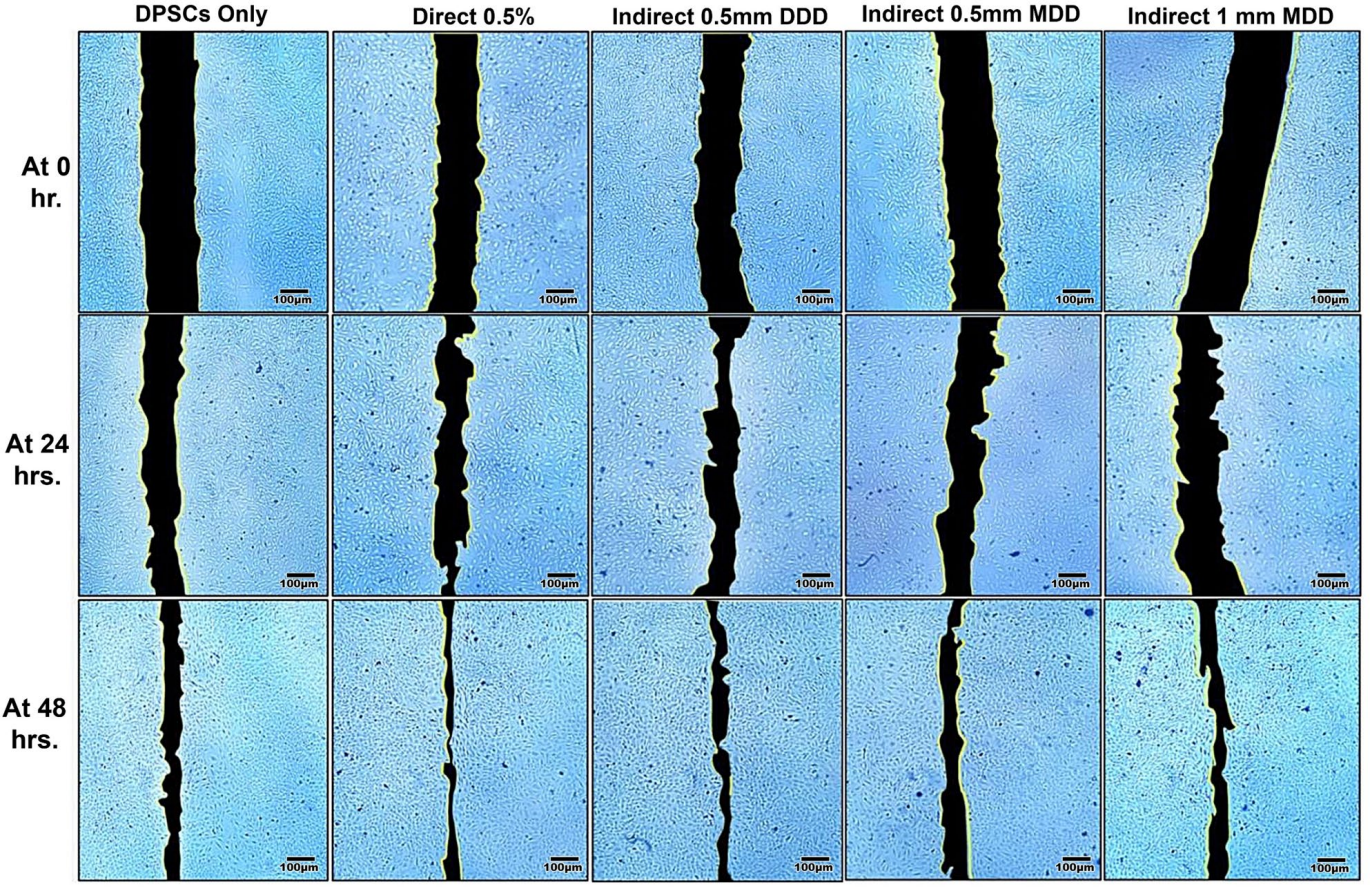
Inverted microscope photomicrogragh shows wound healing in different study groups, (Scale bar = 100 μm, X = 40)

The results of a scratch assay, used to evaluate cell migration and wound healing potential under various experimental conditions over a 48-hour period (Table 5). The data represents the percentage of wound closure area. As expected, the wound area decreased over time in all treated groups, confirming the progression of cell migration. After 24 h, 0.5% direct group, indirect 0.5 mm thick DDD and indirect 0.5 mm thick MDD, as compared with control group, exhibited a non-significant increase of wound closure area (*p* < 0.05). On the other hand, the indirect 1 mm thick MDD group revealed significant initial delay of wound healing after 24 h (*p* < 0.05). Meanwhile, 0.5% direct group at 48 h demonstrated the most pronounced significant accelerated effect (*p* < 0.05) (Fig. 5) (Supplementary file 3).

**Table 5:** Scratch assay results for hDPSCs cells exposed to enzymatic-based CMCR

Scratch Assay					
Groups Time	**DPSCs only**	**Direct**	**Indirect 0.5 mm DDD**	**Indirect 0.5 mm MDD**	**Indirect 1 mm MDD**
After 24 h	35.48 ± 8.78^C^	36.69 ± 8.84^C^	38.57 ± 1.12^C^	35.49 ± 0.00^C^	20.63 ± 6.34^D^
After 48 h	71.51 ± 1.21^AB^	78.78 ± 0.89^A^	74.19 ± 1.03^AB^	65.29 ± 0.84^B^	66.17 ± 0.56^AB^

Test used: Two-way ANOVA test comparing the studied groups in different exposure method and time periods. Values are expressed as means ± SDs. Different letters indicate significant differences between groups (*p* < 0.05). Assay is the experimental unit of our study, number = 3.

### Osteogenic differentiation analysis

The expression levels of DSPP varied notably across the different studied groups. The “indirect 0.5 DDD” group demonstrated a significant increase in the DSPP levels (*p* < 0.05). This was followed by the “indirect 0.5 MDD” group and the “direct” group, with no significant difference in between. Meanwhile, the MDD 1 mm nearly revealed the same results as the control positive group (control + ve) (DPSCs with osteogenic media) (Table 6) (Supplementary file 3).

**Table 6:** Results of DSPP between groups

	Control + ve	direct	Indirect 0.5 mm DDD	Indirect 0.5 mm MDD	Indirect 1 mm MDD	*p*
DSPP	2.14 ± 0.46 ^A^	3.23 ± 0.68 ^A^	5.35 ± 2.01 ^B^	3.56 ± 0.92 ^A^	2.43 ± 0.25 ^A^	0.003*

**One way ANOVA test** was utilized for comparison between the studied groups. The same capital letters indicate non-significant while different indicate significant in the same row at *p* < 0. 05.

## Discussion

Despite substantial improvements in dental technology, dental caries continues to pose a global health concern, impacting individuals of all ages and areas. Traditional caries management approaches often result in unnecessary removal of sound tooth structure, potentially compromising long-term tooth vitality. With the emergence of the minimal intervention dentistry (MID) philosophy, there is a growing emphasis on minimally invasive techniques that aim to arrest caries progression while preserving as much healthy tissue as possible. This underscores the necessity for a comprehensive understanding and evidence-based assessment of minimally invasive caries removal techniques to guarantee optimal patient results and foster long-term oral health maintenance. Minimally invasive approaches in caries management, such as enzymatic-based CMCR, are increasingly favored for their ability to preserve healthy tooth structure and minimize patient discomfort.

Among available CMCR agents, enzymatic-based CMCR has shown clinical promise due to their non-invasive mode of action. However, their interaction with the underlying dental pulp, particularly with DPSCs, remains insufficiently characterized. Given the central role of DPSCs in pulp vitality, regeneration, and dentinogenesis, it is critical to assess the cytotoxicity and biological compatibility of these agents [28]. So, this work investigated the effects of enzymatic-based CMCR on DPSC viability, migration, and osteogenic differentiation, under both direct and indirect exposure conditions. Understanding these interactions will help determine the safety and clinical suitability of enzymatic-CMCR agents in restorative procedures involving deep carious lesions.

The concentration of CMCR used in the MTT assay was selected based on a previous study [29] that evaluated different concentrations (0.5% and 5%). The lower concentration (0.5%) was reported to be biocompatible with minimal cytotoxic effects, whereas the higher concentration (5%) caused significant cell death. Therefore, in the present study, intermediate concentrations (1% and 2%) were chosen to further assess cellular responses within a range close to the biocompatible level, avoiding the cytotoxic effects observed at 5%.

The “Indirect” model in our study was designed to simulate an indirect application of CMCR on pulp tissue through carious dentinal tubules. During treatment of deep carious cavities in clinical situation, a thin layer of caries-affected dentin remains over the pulp after CMCR. In this condition, the pulp tissue does not come into direct contact with the CMCR agent itself, but rather through the overlying dentin that had been previously affected by the chemical softening process. This model was intended to evaluate whether any residual chemical components within the dentinal tubules in treated dentin could influence pulp tissue response when a biocompatible capping material is subsequently applied.

Regarding the MTT results, at 24 h, cell viability decreased significantly with different concentrations of direct exposure groups, however minimal decrease was observed at 0.5% concentration. Meanwhile, cytotoxicity occurred at 1% and 2% concentrations, reducing viability to 61.87 ± 2.71 and 53.5% ± 1.94, respectively. Enzymatic-based CMCR 0.5% showed a trend toward recovery at 48 h, with a viability of 97.5% ± 0.88, indicating biocompatibility over time. At 1% and 2%, enzymatic-based CMCR continued to show significantly lower viability than 0.5% at the same time period. In the indirect exposure groups, all experimental groups had significantly lower cell viability than the control group after 24 h. While DDD 0.5 mm showed higher cell viability (82.7% ± 3.3), it was still significantly different from the control. After 48 h, all indirect exposure groups showed partial recovery. These findings suggested that enzymatic-based CMCR concentration is crucial to DPSCs viability, and lower concentrations may be safer for clinical applications involving dental pulp tissues (direct and indirect pulp exposure).

Our findings agree with Guedes et al., [21] who observed that the enzymatic-based CMCR ™ material caused cytotoxic effects only 50 s post-application, whereas it caused no cytotoxicity after 24 h. This suggests that the gel exerts cytotoxic effects only initially, when it has high activity, without damaging the pulp tissue after 24 h. From the clinical view, assessing the cytotoxic effect is essential in order to show that caries-removing chemicals has to be cautiously utilized because of their potential to cause post-operative pain and pulp necrosis, particularly in deep lesions. Similarly, Bussadori et al., [30] evaluated the effectiveness of enzymatic-based CMCR and success was attained in 13 out of 14 cases.

 [31] conducted an invitro evaluating the transdentinal effect of enzymatic-based CMCRs (namely Brix 3000™ and Papacarie Duo™) on DPCs. They found that indirect application of Papacarie Duo™ for 2 min on dentin discs resulted in cytotoxic effects on DPCs after 24 h, as assessed by the MTT assay. In contrast, the present study expanded the experimental design by introducing a second evaluation period (48 h), assessing the influence of dentin demineralization, varying dentin disc thicknesses, and incorporating direct application of the enzymatic-based CMCR. The results of 0.5% direct exposure group at both time intervals were consistent with those of indirect method via 0.5 mm DDD suggesting that the material exhibits biocompatibility with DPSCs when applied in deep carious cavities, even in cases of pulp exposure.

 [15] reported that enzymatic-based CMCR showed no cytotoxicity on pulp fibroblasts and was effective in treating dental caries. Nevertheless, it only showed cytotoxicity up to 1:4 dilution, indicating that it only reduced cell viability at higher concentration. Enzymatic-based CMCR did not cause cytotoxicity of dental pulp cells at 0.5% concentration [29]. This finding was consistent with [32] who investigated and compared the cytotoxic effects of CMCR on stem cells derived from exfoliated deciduous teeth (SHEDs). Consequently, it has been revealed that enzymatic-based CMCR exhibits no cytotoxic effects [21].

Cell migration plays a pivotal role in the healing of wounded tissues, making it essential for CMCR agents to support this process to preserve dentin integrity. In our study, the effect of enzymatic-based CMCR on cell migration was assessed using the scratch assay at 24- and 48-hour intervals. The findings revealed that 0.5% direct exposure group and 0.5 mm DDD indirect exposure group enhanced wound closure when compared to the control group at 24 h. Notably, the 0.5% direct exposure group exhibited the highest cell migration rate reaching 78.78 ± 0.89% after 48 h, which was significantly higher compared to control group (71.51 ± 1.21%), suggesting a strong regenerative potential associated with direct application of enzymatic-based CMCR on pulp.

The DSPP has been used as mineralization markers for the odontoblast/osteoblast-like differentiation of hDPCs [33]. In the present study, indirect 0.5 mm DDD exposure group exhibited the most significant upregulation of DSPP (5.35 ± 2.01), suggesting a favorable biological response despite the attenuated exposure. This response exceeded that of both the indirect 0.5 mm MDD (3.56 ± 0.92) and direct (3.23 ± 0.68) groups, indicating that low-concentration, indirect exposure under demineralized conditions may mitigate cytotoxicity while preserving or enhancing regenerative potential. Furthermore, enhanced expression in indirect groups supports the hypothesis that soluble components of enzymatic-based CMCR, rather than direct material-cell interaction, may play a central role in modulating gene expression [28].

These results of both cell migration and osteogenic differentiation are in agreement with those of Maru et al. [32], who evaluated the cytotoxic effects of CMCR agents on SHEDs. Their findings also identified 0.5% enzymatic-based CMCR as promoting the highest percentage of viable cells and the greatest extent of cell migration. Furthermore, SHEDs exposed to this concentration showed the highest mRNA expression of osteogenic markers, including alkaline phosphatase after 72 h. Collectively, these results underscore the biocompatibility and bioactivity of enzymatic-based CMCR agents, particularly at 0.5% concentration, in enhancing wound healing and promoting regenerative responses in dental pulp stem cells.

These findings provide preliminary evidence supporting the biocompatibility of enzymatic-based CMCR and its potential role in promoting favorable pulp cell responses when used in minimally invasive treatment protocols. Furthermore, it induces overexpression of odontogenic genes, such as DSPP protein expression in hDPSCs. It is also relevant to perform future research to provide histologic evidence of hard tissue deposition and sealing capability, prior to clinical use.

The present study had some limitations. The study was conducted on cells in a controlled laboratory condition, while clinically there is possible dynamic interaction with direct capping materials used in case of pulp exposure. Such interactions could significantly influence the biological response to the enzymatic-based CMCR agent. Additionally, the study only evaluated the short-term effects of CMCR on DPSCs’ proliferation and osteogenic differentiation. Therefore, further research is recommended to investigate the combined effect of enzymatic-based CMCR and direct capping materials on DPSCs, alongside clinical trials to validate these findings in vivo.

## Conclusion

Enzymatic-based CMCR exhibits favorable biocompatibility, particularly when applied directly on DPSCs at low-concentrations and when applied indirectly using various dentin discs. These findings support this enzymatic caries removal method is potentially safe, effective, and minimally invasive in deep cavities suitable for conservative restorative dentistry.

### Clinical significance

Enzymatic-based CMCR exhibited no adverse effects on dental cells. Enzymatic-based CMCR is considered as an effective, efficient, and non-cytotoxic caries excavation method with minimal cytotoxic effects compared with traditional excavation methods.

## Supplementary Information

Supplementary Material 1.

Supplementary Material 2.

Supplementary Material 3.

## Data Availability

Study data can be provided by the corresponding author when reasonably requested.

## Abbreviations

CMCR: Chemomechanical Caries Removal
DDD: Demineralized Dentin Discs
DMEM: Dulbecco’s Modified Eagle’s Medium
DMSO: Dimethyl Sulfoxide
DPSCs: Dental Pulp Stem Cells
DSPP: Dentin Sialophosphoprotein
EDS: Energy Dispersive X-ray Spectroscopy
EDTA: EthyleneDiamineTetraacetic Acid
FBS: Fetal Bovine Serum
FACS: Fluorescence-Activated Cell Sorting
hDPSCs: Human Dental Pulp Stem Cells
MDD: Mineralized Dentin Discs
MID: Minimally Invasive Dentistry
MTT: 3-(4,5-Dimethylthiazol-2-yl)-2,5-diphenyltetrazolium bromide
PBS: Phosphate Buffered Saline
RT-qPCR: Real-Time Quantitative Polymerase Chain Reaction
SD: Standard Deviation
