# Supplementary figures and images for: Effect of Enzymatic-based chemomechanical caries removal agent on proliferation and osteogenic differentiation of dental pulp stem cells
Source: BMC Oral Health. 2025 Dec 27;26:182. doi: 10.1186/s12903-025-07495-w (PMC12853919; doi:10.1186/s12903-025-07495-w)

**
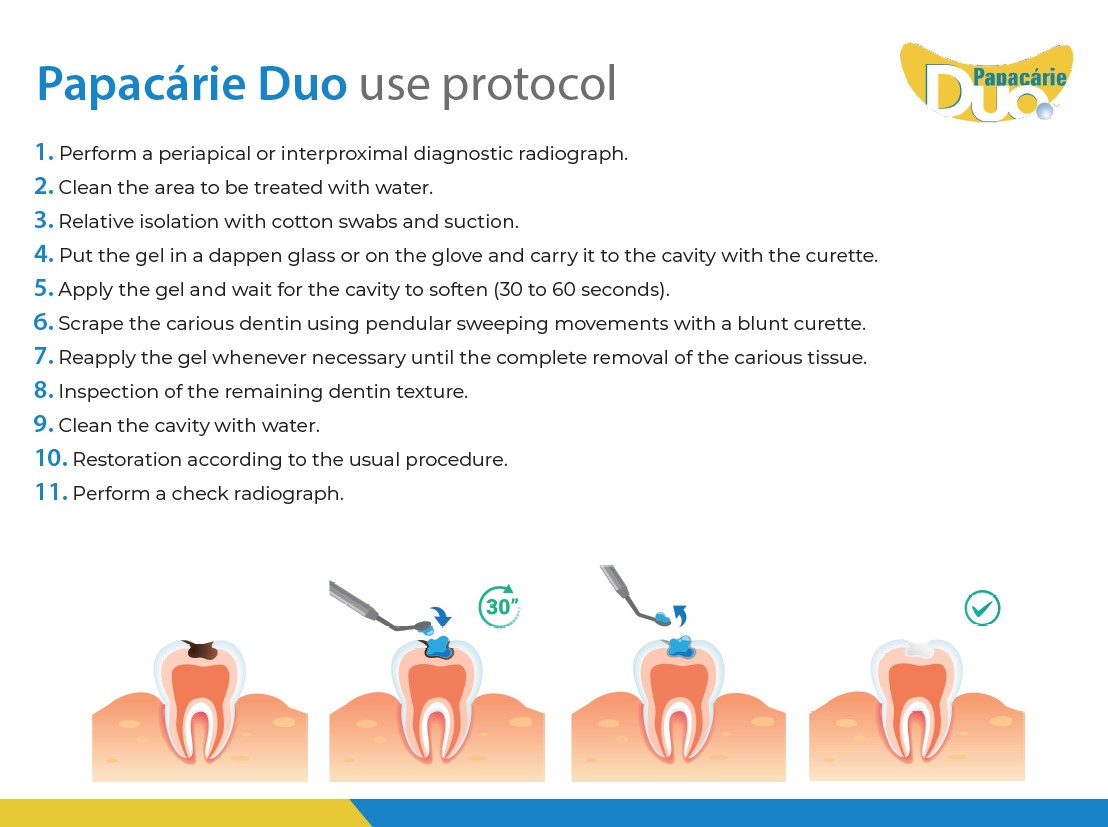
**

Supplement: Supplementary file 2 — Supplementary Material 2. [file 12903_2025_7495_MOESM2_ESM.docx]
