# Supplementary material for: Effect of Enzymatic-based chemomechanical caries removal agent on proliferation and osteogenic differentiation of dental pulp stem cells
Source: BMC Oral Health. 2025 Dec 27;26:182. doi: 10.1186/s12903-025-07495-w (PMC12853919; doi:10.1186/s12903-025-07495-w)
Supplement: Supplementary file 3 [file 12903_2025_7495_MOESM3_ESM.docx]

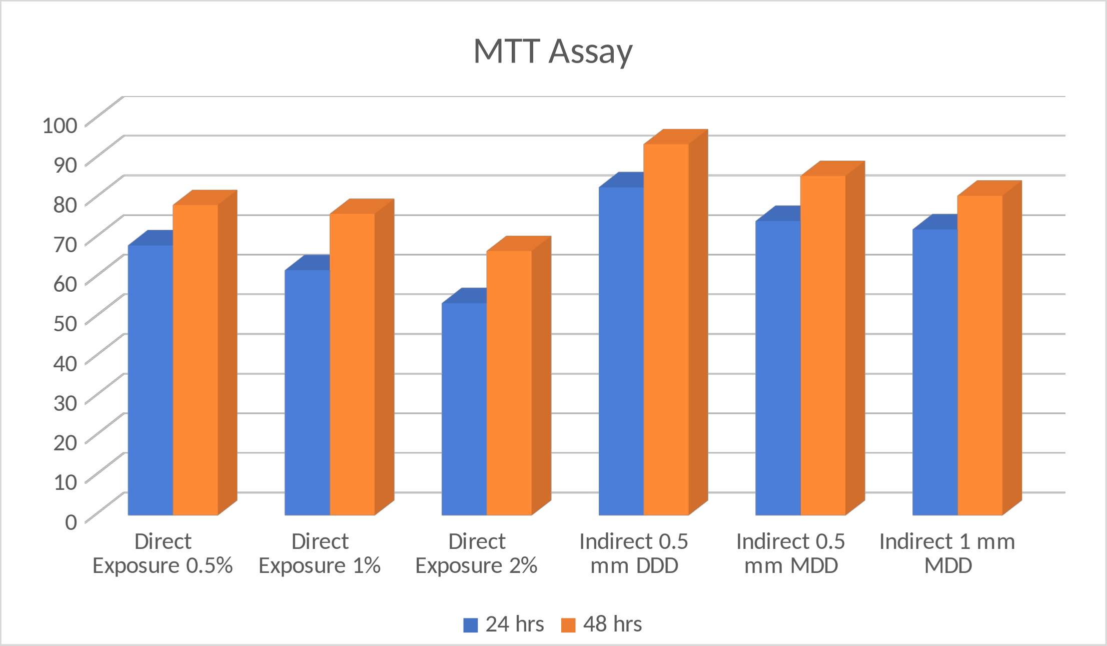
MTT Assay
100
90
80
70
60
50
40
30
20
10
0
Direct Exposure 0.5%
Direct Exposure 1%
Direct Exposure 2%
Indirect 0.5 mm DDD
Indirect 0.5 mm MDD
Indirect 1 mm MDD
24 hrs
48 hrs

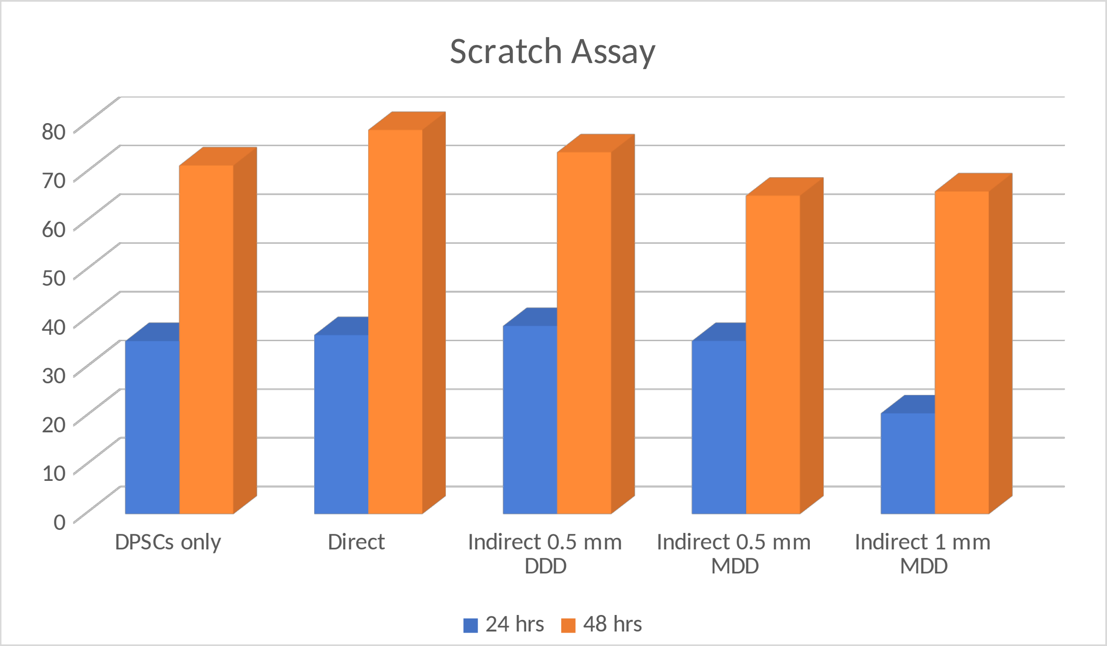
Scratch Assay
80
70
60
50
40
30
20
10
0
DPSCs only
Direct
Indirect 0.5 mm DDD
Indirect 0.5 mm MDD
Indirect 1 mm MDD
24 hrs
48 hrs

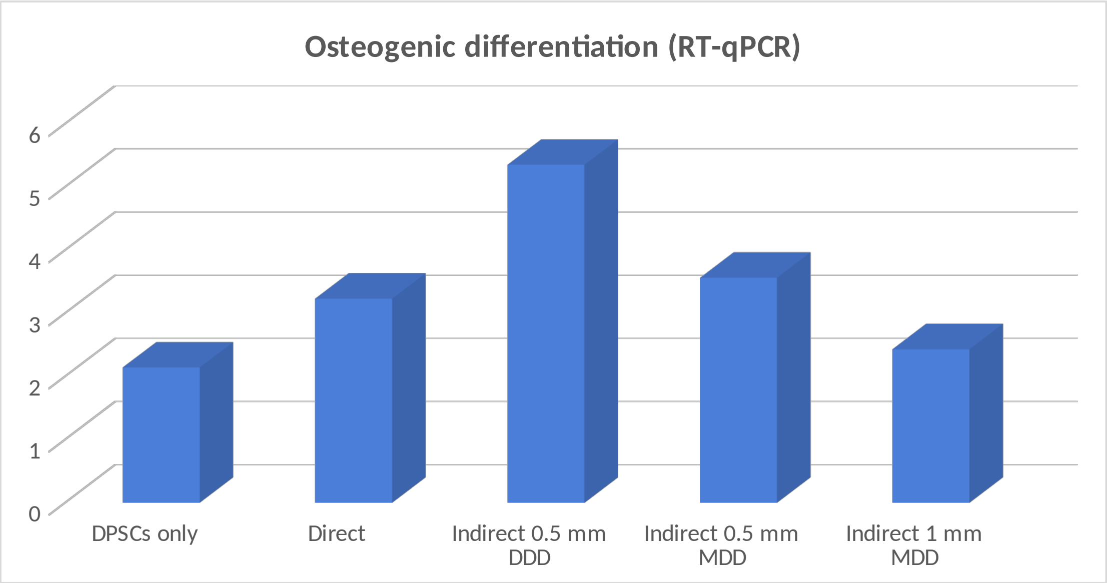

Osteogenic differentiation (RT-qPCR)
0
1
2
3
4
5
6
DPSCs only
Direct
Indirect 0.5 mm DDD
Indirect 0.5 mm MDD
Indirect 1 mm MDD
